# Factors affecting effective communication about sexual and reproductive health issues between parents and adolescents in zandspruit informal settlement, Johannesburg, South Africa

**DOI:** 10.11604/pamj.2016.25.120.9208

**Published:** 2016-10-27

**Authors:** Kegaugetswe Motsomi, Chandra Makanjee, Tariro Basera, Peter Nyasulu

**Affiliations:** 1 School of Health Sciences, Monash South Africa, Ruimsig, Johannesburg, South Africa; 2 Right to Care, Perth Road, Helen Joseph Hospital, Themba Lethu Wing, Westdene; 3 Department of Clinical Sciences, Faculty of Health and Environmental Sciences, Central University of Technology, South Africa; 4 School of Public Health, Faculty of Health Sciences, University of the Witwatersrand, Johannesburg, South Africa

**Keywords:** Adolescent sexuality, parent-child communication, sexual and reproductive health communication, Johannesburg

## Abstract

**Introduction:**

Communication between parents and adolescents regarding sexuality is an important reproductive health topic. Due to complexities associated with adolescent's physiological development, sexuality should be dealt with holistically. This study aimed to investigate factors affecting effective communication between parents and adolescents concerning sexual and reproductive health issues.

**Methods:**

An exploratory qualitative study using the focus group discussions method was done to explore amongst other things; social, cultural and religious barriers to communication. Thematic content analysis was done.

**Results:**

Factors identified included: embarrassment when discussing sexual topics; adolescent misperceptions that guardians want to engage in sexual activities with them; strong belief amongst guardians that reproductive health discussions with adolescents encourages sexual experimentation; belief that adolescents were too young to understand; non-conducive environment for open discussions of sexual and reproductive health matters; cultural and religious beliefs.

**Conclusion:**

In view of these findings, there are still barriers in terms of parent-adolescent engagement on issues related to risks associated with sexual behaviours and erroneous reproductive health choices among adolescents. Therefore, there is a need to encourage engagement by creating neutral platforms facilitated by community healthcare providers and/ or social workers. This will help create awareness and bridge the communication and interaction gap by emphasising the importance of effective engagement among adolescents and their parents on matters related to risks associated with sexual behaviours and erroneous reproductive health choices. Post implantation intervention studies are needed to inform on the outcomes of the intervention.

## Introduction

Globally, an estimated 16 million girls aged 15-19 years give birth each year [1, 2]. Teenage pregnancy rates vary across continents, being lower in Europe (29 per 1 000 girls) where sex is openly discussed, followed by Asia (58 per 1 000 girls) and Africa where rates are highest (130 per 1 000 girls) [3]. Girls aged 15 to 19 years old account for 11% of all childbirths worldwide with 95% occurring in low-and middle-income countries. The highest birth rates among 15 to 19 year olds are in sub-Saharan Africa [4]. Parent-child communication of sexual and reproductive health matters leads to increased awareness on sexual and reproductive matters and is protective for adolescent sexual and reproductive health [5, 6]. Research has found that adolescents have a preference for talking to their parents about sex yet such discussions are rare, and they learn about sexual matters from other sources including mass media and peers [7]. Previous studies have shown that children who discussed sex with their parents were less likely to engage in unsafe sexual behaviours [5, 8, 9]. Closer ties and communication between parents and their children is positively associated with reduced levels of risk taking among adolescents. However, parents do not often communicate about particular topics with their children because they feel embarrassed and experience discomfort when doing so [5, 6, 9]. Studies undertaken in Lesotho and Ethiopia report that as few as 20% of parents discussed sexual matters with their teenage children in contrast to 90% of parents in the USA [6, 8]. Several factors have been previously reported to hinder effective communication between parents and adolescents. A study undertaken in rural Tanzania reported that where communication occurs, it is mainly on same sex basis, usually mother-daughter as adolescents perceived to be strict, intimidating, unapproachable and unavailable [10]. A systematic review of studies on communication about reproductive health issues in sub-Sahara Africa reported that discussions tend to be authoritarian and unidirectional, characterised by vague warnings rather than direct, open discussion [5]. Barriers to having open discussions regarding reproductive health issues are attributed to lack of age-appropriate respectful vocabulary and skills, and, cultural norms and taboos which sanction paternal aunties with the task to discuss sexual and reproductive health matters. Then there is also a perception that children are too young and discussions on reproductive related health matters may promote premarital sex [5–7, 10]. Although several studies have been conducted on barriers to engage or encourage dialogue between parents and adolescents on sexual and reproductive health issues it is not known whether these factors are also relevant in an informal settlement in South Africa. By establishing these factors will contribute to the formulating of strategies to decrease adolescent pregnancies.

## Methods

**Recruitment of study participants:** The study was conducted in Zandspruit informal settlement located within the City of Johannesburg Metropolitan, directly north of Rooderpoort and south of the Cosmo City Development. Zandspruit consists of an estimated 14 500 shacks and is home to over 80 000 people many of whom are unemployed, have inadequate access to clean water, sewage and refuse removal [11]. There is a primary health care clinic, a primary school and no high school in Zandspruit. The informal settlement is characterised by a relatively mobile population hence it consists of a heterogeneous population with many ethnic and linguistic groups. The commonly spoken languages in Zandspruit are Zulu and Tshivenda.

**Study Design :** This was a qualitative descriptive and exploratory study undertaken to explore factors that hinder effective communication between parents and their adolescent children on sexual and reproductive health matters in Zandspruit informal settlement, Johannesburg. Data were collected from 5 focus group interviews. Through a qualitative approach, participants were able to give an in-depth account about their personal feelings, attitudes, opinions and perceptions on communication about sexual and reproductive health matters with their parents. It also increased an understanding of the barriers and facilitators of effective communication about sexual matters within families. We defined a parent as a biological father or mother of the adolescent or a guardian who is responsible for adolescent's welfare. A parent was defined as a biological parent or a guardian who lived with and took care of the adolescent.

**Ethical issues:** Ethical approval was obtained from the Monash University Human Research Ethics Committee number: CF14/2742-2014001529. The confidentiality of the participants were maintained by assigning codes for instance, Focus group2: Participant 1(FG2:P1). Participants were also reassured of their confidentiality and privacy on the information that only the researchers had access. The electronic transcripts were password protected. Their participation was voluntary and was able to withdraw at any time. Participants were also not forced to answer questions that could cause discomfort. A counsellor was also on site in the unexpected event that participants required assistance due to the sensitive nature of the questions.

**Recruitment of study participants:** FIrst contact was made with the Community Development worker and community leaders of Zandspruit settlement. Participants in this study were adolescents and their parents. Purposive sampling was initially used but due to insufficient numbers of participants recruited, the recruitment was achieved through snowball sampling approach using word of mouth i.e. that is adolescents who were contacted informed other adolescents. Participating parents were invited through their children to be recruited in the study. Adolescents (aged 18-19 years) who qualified had to give informed written assent while parents had to give written informed consented.

**Data collection:** Data were collected between August and September 2014. There were 5 focus group discussions FGDs, with a total of 40 participants. Focus group 1 consisted of 8 adolescent girls; Focus group 2 consisted of 8 adolescent boys; Focus group 3 consisted of 8 female parents; Focus group 4 consisted of 8 male parents; and focus group 5 consisted of 8 adolescent mothers. The reason for the particular grouping was to get information rich data as mixing of the groups may have resulted in participants not been open to share due to cultural sensitivity of the study population regarding sexuality. In order to explore the barriers for effective communication between parents and adolescents on sexual and reproductive health issues a focus group discussion guide was used to standardize the focus group discussions. Trained youthful male and female data collectors moderated the FGDs as well as taking notes during the discussions. Young data collectors were selected because they were seamlessly acceptable to targeted adolescents. Male data collectors moderated FGDs composed of male students and females did the same for FGDs of females. During the FGDs participants were also probed on cultural aspects, social values, behaviours and emotions with regard to teenage pregnancies and sexual reproductive issues. All responses were audiotaped and transcribed by professional transcribers who consented to confidentiality. Data saturation occurred when no new data or themes emerged and no new data fitted into the categories already developed from the different focus groups [7].Data collection took place over a period of three weeks. The FGDs were approximately an hour per group thereafter debriefing sessions took place to establish if saturation had been reached. All the quotes were transcribed per verbatim Table 1.

**Table 1:** Sample questions from focus group discussions guide

Questions in adolescent boys and girls FGDs	Questions in female and male parents FGDs	Questions in adolescent mothers FGDs
Do your parents discuss the issues of sexual and reproductive healthwith you?	Do parents discuss the issues of sexual and reproductive health with the children?	Do/did your parents discuss the issues of sexual and reproductive health with you?
What are the things that you think hinder your parents from discussing sex information with you?	In a lot of communities, teenage girls have children what do you think causes this?	Do you think parents should provide sex talk to their children?
What are some of the things that might discourage you to initiate sex talks with your parents?	Which age do you think is appropriate to discuss sex with your child	Which age do you think is appropriate for parents to discuss sex information with their children?
If a young girl living in this community is sexual active, but she does not want to get pregnant. What can she do? Can she initiate the sex talk with her parents? And what content will the parent share with her to ensure that she does fell pregnant?	What issues do you discuss with your children regarding Sexual and Reproductive Health?	In a lot of communities, teenage girls have children, what do you think are the reasons for having children at an early age?
What can be done to get parents involved in discussing sexual matters with you?	Do you think your child gets SRH information from other sources?	What are the implications (both positive and negative) of a teenage child getting pregnant or impregnating someone at an early age?

**Data management and analysis:** The first round of analysis entailed reading and re-reading per verbatim transcripts of what was said by participants to comprehend parent-adolescent communication on sexual and reproductive health matters. Then data were coded and analysed using Nvivo software. At the end of each FGD the research team facilitated debriefing sessions identifying emergent codes and categories in the early stages of data generation to guide subsequent FGDs. Identified categories followed by sub themes were developed on a higher analytical level based on the participants' own language and interpretation to themes.

## Results

The following themes emerged in the focus group discussions: gender related issues, belief that discussing sexual and reproductive health matters encourages sexual experimentation, view that adolescents learn about sexual and reproductive health matters from the media, presence of younger siblings, absent parents, cultural norms and religion.

**Role of transformation from child to adult:** Participants generally perceived that communicating with a child or parent was hindered by the parent-child differences in various facets of understanding and development regarding issues about sexuality. Most parents expressed discomfort discussing sexual issues with their children indicating that they experience embarrassment and they fear being misconstrued by the children that the adult wants to engage in sexual activity with them. Parents felt that rape of children is sometimes orchestrated by their own parents, an indication that nowadays the society does not respect the traditional norms. Their concern is illustrated by the following extract: “…how can a male parent talk to their girl child about sex? The girl child might think that their father has an intention of sleeping with her.” For a boy he will just be embarrassed. (FGD of male parents: FG4). This embarrassment was heightened by the age difference between adolescents and their parents. Adolescents perceived that their parents are too old and conversations on sexual and reproductive health get awkward and prejudicial. This was illustrated through use of quotes directly from adolescents: “For us the age-gap between us and our mothers makes us to be individuals from two different worlds”(FGD of adolescent girls: FG1). In the event of outcomes like pregnancy, adolescents find it hard to reach out to their parents. This was illustrated by the following extract; “There is a big age-gap between us and our mothers and when we fell pregnant they were the last people we informed”(FGD of adolescent mothers: FG5).

**Feeling of shame:** Numerous statements from adolescents revealed that they do not openly communicate with their parents because their parents are strict and they fear that they will be embarrassed and misunderstood. For example, adolescents said: “Oh my God, we don't even want to imagine the shame. When we are watching TV and they broadcast scenes about sex, our fathers more so than our mothers immediately change the channel. We feel embarrassed and it is apparent that both parents are embarrassed too”(FGD of adolescent boys: FG2). “We feel ashamed to talk to our mothers about sex and sexual health issues. Sex is something personal that shouldn't be discussed with our parents. Once you start having this conversation with them they will assume that you have transformed from childhood to adulthood. Even to face our parents after such a discussion will be difficult. We think our mothers will always give us that eye of condemnation thinking that we have started sleeping around with boys”(FGD of adolescent girls: FG1). In addition, female parents commented: “It is very difficult as a woman to discuss anything that concerns sex with your son. We feel embarrassed…”(FGD of female parents: FG3). Most parents cited that it is not appropriate to discuss issues pertaining puberty, condom use, sexual transmitted infections and contraceptives because their children are still too young. As the age of the children increases, parents stated that they will feel more comfortable discussing sexual and reproductive health issues with their children. Parents were found to experience a feeling of shame as discussed below female parents stated: “Our daughters are too young to hear us explain how sexually transmitted infection are contracted. We feel ashamed to share ways of preventing them. We will talk to our daughters when they are old enough”(FGD of female parents: FG3).

**Sexual experimentation ideology:** Parents raised the concern that engaging in discussions on sex and sexual and reproductive health issues will encourage their children to indulge in sex. A good example of this perception is illustrated by the following extract: “Mmmmh…. Children are curious, introducing these ideas in their heads will just lead them to try it out and engage in sex. We can't talk with our children about sex because this will be like we are directing them to engage in sexual activities ”(FGD female parents: FG3). “…As a parent, one should feel ashamed to talk with their children about STIs, condom use, and pregnancy prevention methods because these children are still too young to know all of these sensitive issues” In addition “Sexually transmitted infections are diseases for adults we really don't see any point in discussing such issues with our children, they will know about all of this when they become adults”(FGD of male parents: FG4).

**Media as a source of information :** It was found that parents believed that their children find the required reproductive health information from the media emphasising that media puts their children at high risk of engaging in risky sexual behaviours. They stated that their children prefer to watch televisions, browse the internet than ask them questions about issues of sex and sexual health. During the focus group discussions, male parents shared their anxieties about these issues and said: “Our children say they live in an era which is advanced in terms of science and technology where a child can get information from brochures, televisions, internets and journals, that is why they do not see any reason to talk to anyone about this issues”(FGD of male parents: FG4). “Because of the internet our daughters think they can just google everything. Do you think that our children don't know about how pregnancy, STIs and HIV/AIDS is obtained and how to prevent them? We know they know because of science and technology that is why it doesn't carry weight to talk to them and we get embarrassed too talking about these privates issues”(FGD of female parents: FG3). Parents who lead busy lives tend to be a barrier to communication between them and their adolescents concerning sexual and reproductive health issues. Adolescents feel disconnected from their parents due to the fact that they are always busy with work or never home. Adolescents highlighted the need for direct communication however their parents just give them reading materials. “…parents don't understand that magazines cannot replace them, when some of us first had our periods, we were alone at home and there was blood everywhere and we freaked out and called our mothers only to be told they are busy. Ever since this experience happened, we told ourselves that we don't need them when such incidents happen”(FDG of adolescent girls: FG1). Parental behaviour was perceived as a major determinant of effective personal communication between adolescents and their parents. Children feel at ease to talk to their parents if they are welcoming and are present in their life as they grow up. Their frustration in discussing SRH issues with fathers is well illustrated by the following extract; “Our fathers drink like a fish and they are never home, they only come home when there is a funeral…laughing!!!, when we need them, they are nowhere to be found so we have resorted to consulting friends and getting information from the media when something is unclear to us”(FGD of adolescent boys: FG2).

**Presence of younger siblings in the home:** Another barrier for not discussing sexual matters openly within the family was the presence of younger siblings. One teenage boy stated that usually conversations are interrupted by his younger siblings. When asked about contextual factors that hindered communication about sexual matters, parents felt that conversations about sexual matters were restricted because the content would be inappropriate in front of younger siblings. : “We get a chance to talk about these issues during the night time. We have half-an-hour when younger children have gone to bed, so we generally talk about things like these at that time” (FGD of female parents: FG3). To overcome such challenges in communication, parents prefer to talk to their older children about sex and sexual health matters during the time that the younger siblings are not present. “We are sometimes restricted on what we discuss because of our younger children. There is no way we could talk about some things with them being around so we think sometimes the moment has passed if they mention something and we can´t elaborate on it because our younger children and their friends will be in the house” (FGD of male parents: FG4) .

**Culture:** Culture and cultural beliefs act as an inhibiter or better still a deviant in mediating and addressing issues of sex and sexual and reproductive health. Participants explained that it is a taboo to talk openly about sexual and reproductive issues in their cultures. They explained that senior or elderly members of the family are the ones responsible for talking about sexual and reproductive health issues with children as evidenced by these sentiments: “In our culture issues of sexual and reproductive health are done through initiation schools not by parents. We are embarrassed to talk to our daughters about menstruation because we learnt about this from an initiation school not from our mothers” (FGD of adolescent girls: FG5). Other examples, shared by participants were: “Our culture does not allow us as parents to directly talk to our children about issues of sexual and reproductive health, it is the responsibility of senior members of the family”(FGD of male parents: FG4). “Matters to do with sex are traditionally a taboo in our culture, such are private subjects, not befitting public discourse”(FGD of female parents: FG3).

**Religious beliefs:** Parents mentioned that their religious beliefs guide them on what to discuss with their children. They focused on teaching their daughters about the virtues of virginity and that they should forget about sexual activities until they got married. Hence this is the barrier that makes them fail to talk with their children about issues of sexual and reproductive health. This was evident from the narratives below: “Christianity helps us in communicating with our children as it does not allow adultery which is a sin against God. Our religion also prohibits condom use, so we can't tell our children to use condoms since we don't like our children to use condoms as it is not the will of God”(FGD of male parents: FG4). “It is a disgrace to talk with our children of any gender about sexual and reproductive health, even our religion prohibit us from talking about these issues. This is the responsibility of seniors who are not the parents of the child” (FGD of male parents: FG4) Table 2.

**Table 2:** Emerging themes

Theme	Description
1.Role of transformation from child to adult	Some adolescents felt that their parents were too old to understand their way of life, whereas parents felts that discussing such issues implied meant the adolescents were now adults
2.Feeling of shame	Parents- adolescents felt embarrassed to discuss sex and reproductive health matters
3.Belief that discussing sexual and reproductive health matters encourages sexual experimentation	Difficulty in communicating with adolescents as it was understood as facilitating early sexual debut
4.Media as the source or trigger to risky access to knowledge	Adolescents prefer to learn about sexual matters from the media
5.Presence of younger siblings in the house	Need for a conducive environment to discuss sexual and reproductive health matters
6.Cultural roles and responsibility	Specific adult family members have the responsibility of communicating to adolescents about issues related to sexual and reproductive health matters
7.Religion	As an enabler/disabler of preventative measures

## Discussion

The most critical finding of this study was that adolescents were keen to communicate with their parents about sexual and reproductive matters and vice versa. However, it was found that when an interaction occurred parents presented themselves as authoritative thus making their children embarrassed to discuss sexual matters with them. Similar findings were reported in a study conducted in rural Tanzania [12]. For both parents and their adolescent children gender played an influential role when parent-child effective interaction took place regarding sexual and reproductive health related issues. One of the reasons from the parent perspective was the sensitivity and emphasis on sexuality in the African context. It was also highlighted that adolescents were more comfortable to discuss sexual and reproductive health issues with senior members of the family like their grandparents. This approach stemmed from the fact that grandparents tend to take a more less stricter role hence develop an environment that allows their grandchildren to be free and discuss sensitive issues with them. Adolescents regard their parents as being unapproachable and they feared being misunderstood as well as being questioned on private matters. Such factors may undermine the enablers to communicate effectively with parents who are a key source of information. The role of the parents and adolescents in the society resulted in them having a different worldview, with parents strongly attached to cultural beliefs which inhibit them from holding discussions on sexual and reproductive health issues with their adolescent children. These findings concur with previous studies where parents were more conservative to give sex education to adolescents [13]. Parents were of the opinion that adolescents prefer getting information about sexual and reproductive health from the media such as television and internet hence they were knowledgeable in sexual and reproductive related issues. This is similar to previous findings [14], therefore diminishes the parents' role as the primary source of information. The responsibility is relegated to other sources, which are too characterized by information gaps [15].

The age of these children was identified as the most common barrier to communication about sex because parents are comfortable to initiate discussions with adolescents. It was reported that as the age of the child increases parents will feel more comfortable to speak about sex to their children. The findings are similar to a previous study which showed parents speak less to younger adolescents because they believe it is not yet appropriate to talk about sex at such a young age [16]. One study suggested that parents only initiated communication as a means of protection once they discovered the teenager to be sexually active [17]. Sexual and reproductive health issues are culturally sensitive hence are often not openly discussed between parents and children. This finding is consistent with other studies where cultural beliefs are still intact and continues to inhibit parents from discussing sexual matters with their children [18, 19]. Cultural beliefs and taboos about sexuality are deeply rooted in parents' lives and hinder communication. Religion also played a role in inhibiting communication. Parents from some religious background such as the Christian faith found it very difficult to recommend the use of contraceptives to their children, let alone to talk about engaging in protected-premarital sex as it was at odds with morality and religious values as reported in other studies [19]. There were a few limitations regarding the research design as it was exploratory in nature not underpinned by a research philosophical framework such as a phenomenological or an ethnographic approach to guide regarding data interpretation and analysis. However, as it was the first attempt to establish factors a descriptive approach sufficed. The strength of this study lies in that the findings of the study contributes to studies of a similar nature but more so in the adolescent category.

## Conclusion

This study found that gender differences and traditional or cultural norms inhibit effective parent-child communication concerning sexual and reproductive health matters. Parent-child communication about sexual and reproductive health issues is important in reducing risky sexual behaviours and consequences associated with such behaviours among adolescents. For this reason, there is need to tailor interventions to accommodate both worlds and finding the middle ground between parents and children through taking into account the roles and responsibilities of parents and their adolescent children while promoting cultural integration of traditional and western norms as a way of encouraging effective parent-child communication.

### What is known about this topic

- Parent-child communication on sexual reproductive health has been identified as a protective factor for adolescent sexual and reproductive health, including HIV infection;
- Sensitive issues such as explanation on condom use, acquisition of HIV/AIDS and STIs as well as the child's physical development are rarely discussed because of lack of knowledge and cultural norms that restrict interaction between opposite sex;
- When SR discussions occur they are mainly on same sex basis and they tend to be authoritarian and uni-directional.

### What this study adds

- Gender differences and cultural norms are major hindrances to effective communication about sexual and reproductive health issues in Zandspruit, South Africa;
- There is need to find ways to effectively facilitate sexual and reproductive health matters to children through their families;
- Public education should be strengthened to encourage communication between parents and their children while promoting cultural integration of traditional and western norms.
